# Synovial glucose and serum-to-synovial-glucose ratio perform better than other biomarkers for the diagnosis of acute postoperative prosthetic knee infection

**DOI:** 10.5194/jbji-10-41-2025

**Published:** 2025-03-04

**Authors:** Marta Sabater-Martos, Oscar Garcia, Laia Boadas, Laura Morata, Alex Soriano, Juan Carlos Martínez-Pastor

**Affiliations:** 1 Orthopedic and Traumatology Department, Clínic Barcelona, Carrer Villarroel 170, 08036 Barcelona, Spain; 2 Department of Infectious Diseases, Clínic Barcelona, Carrer Villarroel 170, 08036 Barcelona, Spain; 3 IDIBAPS (Institut d'Investigacions Biomèdiques Agustí-Pi Sunyer, University of Barcelona, Barcelona, Spain; 4 CIBERINF (CIBER for infectious diseases), Barcelona, Spain

## Abstract

**Introduction**: In native septic arthritis, synovial glucose is a well-established diagnostic marker. However, its diagnostic utility in periprosthetic joint infection (PJI) remains unexplored. Given the diagnostic challenges of acute postoperative PJI, we hypothesized that synovial glucose could serve as a valuable biomarker and aimed to evaluate its diagnostic accuracy.

**Material and methods**:  This is a retrospective diagnostic study in acute postoperative PJI in total knee arthroplasty (TKA). We reviewed all TKA surgeries performed in the past 10 years and cross-checked those patients that consulted to our emergency room during the first 90 d after TKA surgery for knee-related symptoms. We calculated the serum-to-synovial-glucose ratio for each patient (serum-to-synovial-glucose ratio = [(serological glucose – synovial glucose) / serological glucose]), and we formed the receiver operating characteristic (ROC) curves for synovial glucose, serum-to-synovial-glucose ratio, serum C-reactive protein (CRP), synovial white blood cell (s-WBC) count, and polymorphonuclear cell percentage (PMN%); then we extracted the optimal cutoff values.

**Results**: The optimal cutoffs for diagnosing acute postoperative PJI were < 44 mg dL^−1^ for synovial glucose and > 0.69 for serum-to-synovial-glucose ratio. The area under the curve (AUC) values were 0.861 and 0.889, respectively. ROC curves for serum CRP, s-WBC count, and PMN% showed AUC values of 0.69, 0.714, and 0.66, respectively. The combined ROC curve analysis for serum CRP, s-WBC count, and PMN% showed an AUC of 0.722. When adding synovial glucose, the AUC was 0.859 and with serum-to-synovial-glucose ratio we achieved an AUC of 0.876.

**Conclusion**: Synovial glucose and serum-to-synovial-glucose ratio demonstrated good diagnostic potential for acute postoperative PJI following TKA. These biomarkers exhibited superior accuracy compared to the combination of serum CRP, s-WBC count, and PMN%.

## 1 Introduction

Knee arthroplasty replacement is one of the most performed procedures in the field of orthopedics. Unfortunately, infection remains a significant complication in prosthetic surgery, affecting approximately 0.5 % to 2 % of patients, with a higher incidence of 10 % in revision surgeries (Bauer et al., 2006; Kurtz et al., 2010; Tande and Patel, 2014; Zimmerli et al., 2004). Over the past decades, there has been a significant increase in research on periprosthetic joint infection (PJI), leading to the development of consensus guidelines for optimal diagnosis (Parvizi et al., 2013, 2018; Signore et al., 2019; Workgroup and Society, 2011). Assessing synovial fluid biomarkers such as white blood cell (WBC) counts and the polymorphonuclear cell percentage (PMN%) has proven valuable in diagnosing chronic PJI. However, recent studies suggest that current cutoff values for WBC and PMN% may not be accurate for diagnosing acute postoperative PJI, indicating the need for higher cutoff values (Bedair et al., 2011; Fernández-Sampedro et al., 2017).

Research findings have indicated that the levels of synovial fluid WBC count and PMN% can exhibit variability over time, regardless of whether an infection is present or not (Christensen et al., 2013). Consequently, the diagnosis of acute postoperative PJI is challenging. Even though there are defined thresholds for synovial fluid analysis, these do not always provide definitive diagnostics. Clinicians often rely on their clinical suspicion or wait for culture results, which delays initiating appropriate treatment. Therefore, having an easy and rapid test can help clinicians in the diagnosis of early postoperative PJI.

In native septic arthritis, the role of synovial glucose in the diagnostic process has been well established (Brennan and Hsu, 2012; Elsissy et al., 2020). In the case of PJI, the investigation of this biomarker is still lacking. We anticipated that replaced knees would exhibit similar synovial glucose patterns to those observed in native septic arthritis. Hence, it was deemed necessary to conduct a study to determine the diagnostic accuracy of synovial glucose and serum-to-synovial-glucose ratio in acute postoperative PJI. The objectives of this study were (1) to identify the optimal cutoff value for synovial glucose and the serum-to-synovial-glucose ratio; (2) to evaluate the diagnostic accuracy of both tests; (3) to compare to serum C-reactive protein (CRP), synovial WBC, and synovial PMN%; and (4) to establish a combined receiver operating characteristic (ROC) curve for serum C-reactive protein (CRP), synovial WBC count, PMN%, and synovial glucose.

## 2 Material and methods

This study was approved by our institutional ethics committee (CEIM: HCB/2020/0517). This is a retrospective cohort study including consecutive patients diagnosed with acute postoperative PJI in total knee arthroplasty (TKA). We defined acute postoperative PJI as those infections diagnosed during the first 90 d after index surgery (Parvizi and Gehrke, 2014). Diagnosis was reached with two positive cultures with identical microorganism or when three minor criteria of the Musculoskeletal Infection Society (MSIS) were reached (Parvizi and Gehrke, 2014).

We reviewed all TKA surgeries for osteoarthritis performed from 1 January 2010 to 31 December 2019 and cross-checked those patients that consulted to our emergency room during the first 90 d after TKA surgery for knee-related symptoms. We included patients who had undergone a joint fluid aspiration. We classified them as septic cases if they had a positive culture or at least three minor criteria. Aseptic cases were patients with suspected PJI that had a knee arthrocentesis within 90 d after TKA with negative cultures and less than three minor criteria (Goswami et al., 2018). To maintain the focus of the study on primary knee replacement surgeries, patients who underwent non-primary knee replacements were excluded from the analysis.

We collected demographic data, infective organism, time to aspiration, serum concentration of serum CRP, glucose, red blood cells in blood, and leucocyte counts; we also reviewed synovial concentration of glucose, synovial red blood cell count, WBC count, and PMN%. We also reviewed knee-related symptoms that lead to knee arthrocentesis; we divided those symptoms into two categories: patients presenting with erythema, swelling, or pain and patients showing wound problems. We defined wound problems as the presence of dehiscence, wound leakage after the first week, and/or skin necrosis.

###  Material and methods

Demographic data were compared between groups using the chi-squared or *t* test where applicable as they followed normality. Mean laboratory values were compared between groups using the *t* test for non-parametric variables as they did not follow normality. From data extracted on serological glucose and synovial values, we calculated the serum-to-synovial-glucose ratio for each patient (serum-to-synovial-glucose ratio = [(serological glucose – synovial glucose) / serological glucose]). We calculated the receiver operating characteristic (ROC) curve for synovial glucose and serum-to-synovial-glucose ratio and extracted the optimal cutoff values as the value that maximized the Youden index. We calculated sensitivity, specificity, positive predictive value (PPV), negative predictive value (NPV), and accuracy for both tests. We used the area under the curve (AUC) to assess each tests overall performance and compared both curves using DeLong's test (Akobeng and Akobeng, 2007). In addition, we calculated the receiver operating characteristic (ROC) curve for serum CRP, synovial WBC count, and PMN%, and we extracted the optimal cutoff value for our data as the value that maximized the Youden index. We assessed the overall performance of each of these curves with synovial glucose and serum-to-synovial-glucose ratio using DeLong's test. The statistical performances of combined tests for serum CRP, synovial WBC count, PMN%, synovial glucose, and serum-to-synovial-glucose ratio were also calculated using a combined ROC curve. We also searched for a correlation between synovial glucose as well as serum-to-synovial-glucose ratio and microbiology and knee-related symptoms using the Spearman correlation coefficient. We set statistical significance at 5 % alpha level and performed all statistical analysis with Jamovi (The Jamovi project, 2024).

## 3 Results

We identified 165 patients who sought medical attention at the emergency department within the first 90 d after TKA. After applying our inclusion and exclusion criteria, we excluded 87 patients due to presenting with issues unrelated to the operated knee, leaving us with a final sample size of 78 patients. No patients received antibiotics previous to joint aspiration. Among these, 32 patients were classified as septic cases, while the remaining 46 were categorized as aseptic cases.

The mean (SD) age of the patients in our study was 72.8 (9.8) years. The mean (SD) time interval between the TKA surgery and joint fluid aspiration was 29.7 (19.8) d. In terms of demographic characteristics, septic and aseptic cases had similar ages, sex distribution, laterality, and comorbidities, except for the time lapse from index surgery to joint fluid aspiration. The septic cases had a significantly longer time to aspiration compared to the aseptic cases (35.4 d in infected cases vs. 25.8 d in controls; *p*= 0.034) (Table 1).

**Table 1:**
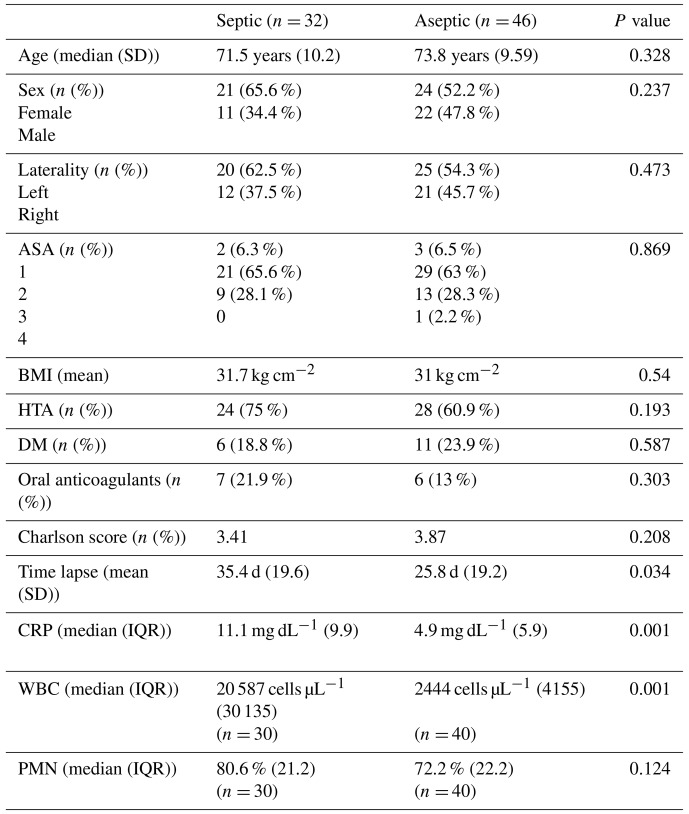
**Table 1**Differences between septic and aseptic groups.

ASA: American Society of Anesthesiologists; BMI: body mass index; HTA: hypertension; DM: diabetes mellitus; CRP: C-reactive protein; WBC: white blood cell count; PMN: polymorphonuclear; IQR: interquartile range.

We also conducted a comparison of other blood and synovial laboratory values. No significant differences were observed between septic and aseptic cases in terms of red blood cell count in blood, serum glucose concentration, and synovial red blood cell count. However, when we examined tests that are commonly associated with acute infections, such as serum CRP blood leucocyte counts and synovial WBC count, we found significant differences between infected septic and aseptic patients (*p*= 0.001, for all comparisons). Additionally, although not statistically significant (*p*= 0.124), septic cases also exhibited higher synovial PMN% levels (Table 1). Furthermore, we observed notable differences in synovial glucose levels between septic and aseptic cases (*p* < 0.001). The median synovial glucose level was 23 mg dL^−1^ (IQR 41.8) in the septic cases, while it was 79 mg dL^−1^ (IQR 54.5) in the aseptic cases. Similarly, the serum-to-synovial-glucose ratio differed significantly between the two groups, with a value of 0.77 (IQR 0.31) in septic cases and 0.31 (IQR 0.33) in aseptic cases (*p* < 0.001). 

### 3.1 Results

**Figure 1:**
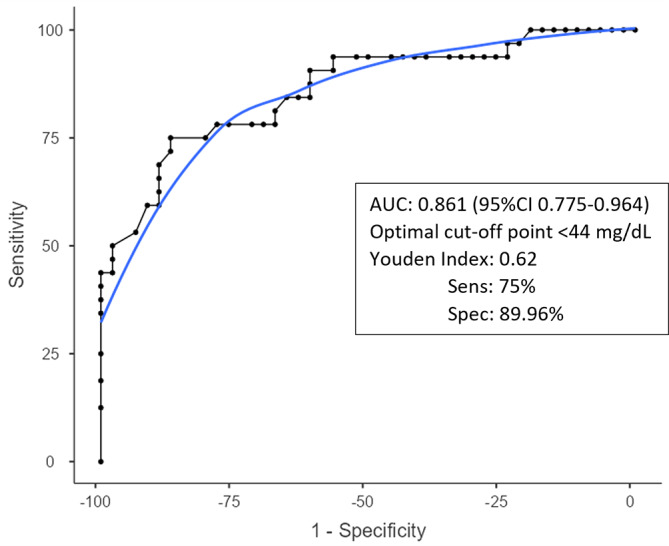
**Figure 1**Synovial glucose ROC curve.

**Figure 2:**
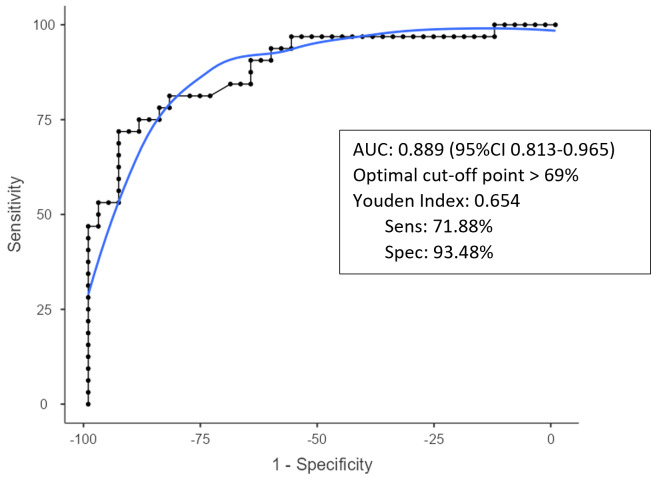
**Figure 2**Serum-to-synovial-glucose ratio ROC curve.

The optimal cutoff value for diagnosing acute postoperative PJI for synovial glucose was found to be < 44 mg dL^−1^, with an AUC of 0.861 (95 % CIconfidence interval 0.775–0.964), a sensitivity of 75 %, and specificity of 86.9 % (Fig. 1, Table 2). For the serum-to-synovial-glucose ratio, the optimal cutoff value was determined to be > 0.69, with an AUC of 0.889 (95 % CI 0.813–0.965), sensitivity of 71.88 %, and specificity of 93.98 % (Fig. 2, Table 2). DeLong's test was used to compare both ROC curves, but no significant differences were found (p= *0.153*).

**Table 2:**
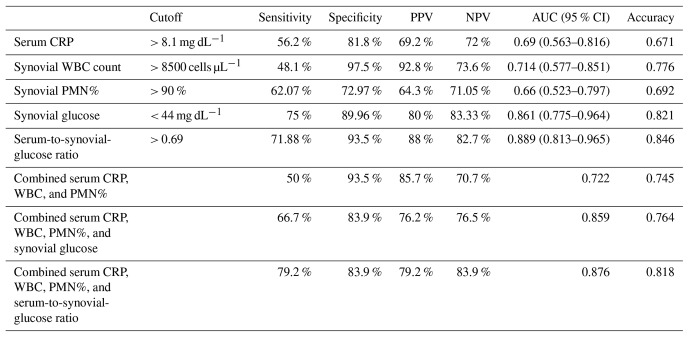
**Table 2**Diagnostic laboratory test performance at optimal cutoff values.

PPV: positive predictive value; NPV: negative predictive value; AUC: area under the curve; CRP: C reactive protein; WBC: white blood cells; PMN%: polymorphonuclear percentage

**Figure 3:**
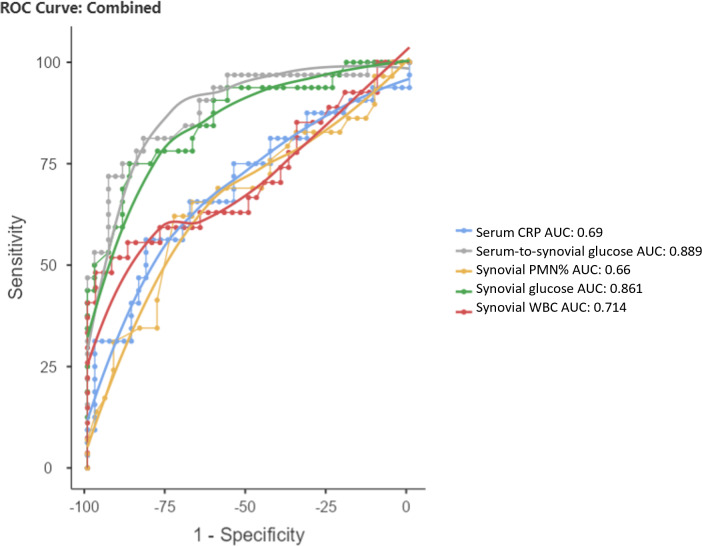
**Figure 3**Serum CRP, synovial WBC count, and PMN% ROC curves.

**Figure 4:**
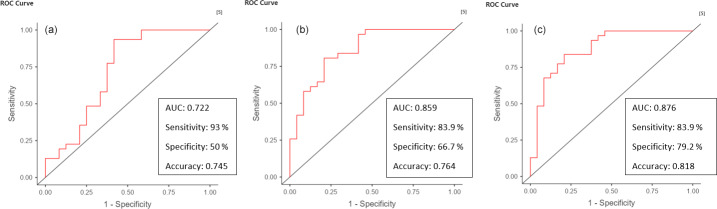
**Figure 4**Combined ROC curves for **(a)** serum CRP, synovial WBC count, and synovial PMN%. **(b)** Serum CRP, synovial WBC count, synovial PMN%, and synovial glucose. **(c)** Serum CRP, synovial WBC count, synovial PMN%, and serum-to-synovial-glucose ratio.

The ROC curves for serum CRP, synovial WBC count, and PMN% showed AUC values of 0.69 (95 % CI 0.563–0.816), 0.714 (95 % CI 0.577–0.851), and 0.66 (95 % CI 0.523–0.797), respectively (Fig. 3). When combined, the ROC curve analysis for serum CRP, synovial WBC count, and PMN% yielded an AUC of 0.722 (Fig. 4a). The addition of synovial glucose increased the AUC to 0.859, and with serum-to-synovial-glucose ratio we achieved an AUC of 0.876 (Fig. 4b and c). DeLong's test was used to compare synovial glucose and serum-to-synovial-glucose ratio ROC curves with all other values; all showed statistical differences expect synovial glucose with WBC count (Table 3).

**Table 3:**
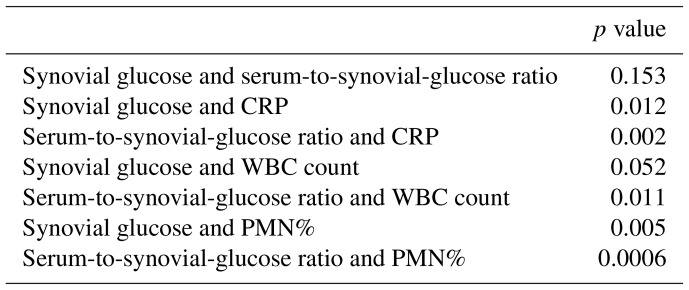
**Table 3**ROC curve correlation.

CRP: C-reactive protein; WBC: white blood cell; PMN%: polymorphonuclear percentage

### 3.2 Correlation

Thirty septic cases showed positive cultures. The vast majority of infections were monomicrobial (87 %). The most common microorganism was *Staphylococcus aureus* (35.1 %), followed by coagulase-negative staphylococci (CoNS) (10.8 %), and the remaining 54 % were comprising various gram-negative bacteria (GNB) (including *Pseudomonas aeruginosa*, *Escherichia coli,* *Serratia marcescens, Klebsiella pneumoniae, Enterobacter cloacae, Morganella morganii, Bacteroides fragilis*, and *Proteus mirabilis*). Polymicrobial infections exhibited synovial glucose levels above the calculated cutoff value (44 mg dL^−1^) and a serum-to-synovial-glucose ratio below the threshold (0.69). However, no significant correlation was found between synovial glucose or serum-to-synovial-glucose ratio and a specific pathogen, with Spearman correlation coefficients of 0.229 and −0.231, respectively (*p*= 0.223 and 0.219).

**Figure 5:**
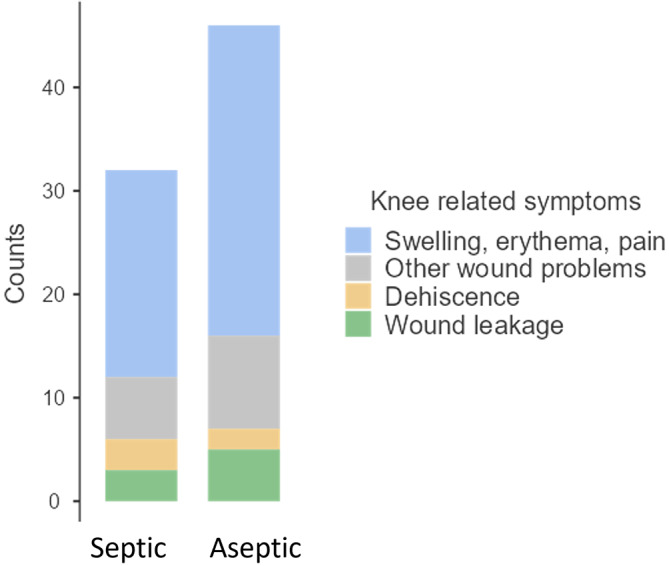
**Figure 5**Knee-related symptom distribution between infected cases and controls.

The reasons to perform an arthrocentesis was erythema, swelling, or pain in 50 patients (64.1 %) and wound problems in 28 patients (35.9 %). Distribution between septic and aseptic cases can be seen in Fig. 5. Septic cases with erythema, swelling, or pain exhibited a median (IQR) synovial glucose level of 9.1 (8.05) mg dL^−1^ and median (IQR) serum-to-synovial-glucose ratio of 0.92 (0.25). Septic cases with wound problems showed a higher median (IQR) synovial glucose level (30.5 (26.65) mg dL^−1^) and a lower median (IQR) serum-to-synovial-glucose ratio (0.713 (0.204)); differences in both groups were not statistically significant (*p*= 0.46 and *p*= 0.36, respectively). Both groups presented median values of synovial glucose levels below the cutoff (< 44 mg dL^−1^) and a serum-to-synovial-glucose ratio above the cutoff (> 0.69). Three patients with erythema, swelling, or pain presented above the synovial glucose cutoff values (44 mg dL^−1^) and below the serum-to-synovial-glucose ratio (0.69). All of them were diagnosed with prosthetic infection with two or more positive cultures for the same microorganism, and none of them presented with WBC count or PMN% elevation. Five patients with wound problems presented a synovial glucose level above the cutoff (44 mg dL^−1^): two of them were diabetic and presented a serum-to-synovial-glucose ratio above the cutoff (0.69). The other three patients were also diagnosed with prosthetic infection with two or more positive cultures for the same microorganism, and none of them presented with WBC count or PMN% elevation.

## 4 Discussion

PJI is a serious complication that can occur following a total joint arthroplasty and can lead to severe consequences if not promptly identified and treated. The Musculoskeletal Infection Society (MSIS) introduced diagnostic criteria for PJI in 2011, which have undergone several updates until the international consensus meeting (ICM) in 2018 (Parvizi et al., 2013, 2018; Workgroup and Society, 2011). We decided to use MSIS criteria due to a lack of some tests performed in newer classifications such us ICM 2018 or EBJIS 2019 (Goswami et al., 2018; Mcnally et al., 2021). While the majority of the literature focuses on chronic PJI, several studies have demonstrated different laboratory values for acute and chronic PJI (Goswami et al., 2018; Kim et al., 2017a; Sukhonthamarn et al., 2020).

To the best of our knowledge, this is the first study to evaluate synovial glucose as a diagnostic tool for acute postoperative PJI. Synovial glucose is widely used in other septic conditions such as meningitis and peritonitis (Davis, 2018; Krastev et al., 2013). Low glucose levels have also been employed as a diagnostic indicator in septic native arthritis; some studies use the serum-to-synovial-glucose ratio < 0.5 (Carpenter et al., 2011), while others directly use the synovial fluid glucose threshold of < 1.5 mmol L^−1^ (15 mg dL^−1^) (Söderquist et al., 1998). In our study, we identified the optimal cutoff values for synovial glucose as < 44 mg dL^−1^ and > 0.69 for serum-to-synovial-glucose ratio. These values are similar to those reported by Kinugasa et al. (2020), who described levels below 40 mg dL^−1^ as suspicious for septic arthritis in native joints among children.

In our study, we conducted ROC curve analysis for both synovial glucose and the serum-to-synovial-glucose ratio. Both curves demonstrated high accuracy with no statistical differences. However, when evaluating the diagnostic accuracy of combined serum CRP, synovial WBC count, and PMN%, the serum-to-synovial-glucose ratio seems to perform better than synovial glucose (AUC values of 0.876 vs. 0.859). Our study demonstrates that the serum-to-synovial-glucose ratio exhibits better accuracy for diagnosis and stronger correlation. Therefore, whenever possible, we recommend using the serum-to-synovial-glucose ratio, though when serum glucose is not available synovial glucose can also be used.

We also assessed the performance of serum CRP, synovial WBC count, and PMN%. Consistent with other studies, we found that synovial WBC exhibited the highest accuracy among the three tests (Bedair et al., 2011; Kim et al., 2017b; Sukhonthamarn et al., 2020; Yi et al., 2014). However, our ROC curves demonstrated lower performance compared to these studies for all three tests. Nevertheless, the accuracy increased to an AUC of 0.722, and further improvement was observed when synovial glucose or serum-to-synovial-glucose ratio were added (AUC values of 0.861 and 0.889, respectively).

Acute postoperative infections could be the consequence of contamination during surgery or from the manipulation of a delayed wound closure (i.e., resuturing, taking samples, and/or application of advanced therapies when closure is delayed) in the postoperative period. In the first case, the infection is usually monomicrobial, and the infection progresses from the joint space to outside, being a sign of septic arthritis (pain, erythema, swelling). In contrast, those infections that result from exogenous contamination are more often caused by polymicrobial flora that penetrates to the joint space, usually presenting with less septic arthritis signs unless the diagnosis is significantly delayed (Benito et al., 2019). This could partially explain the poor performance of synovial glucose observed in the second group of infections. In our results, we found four patients with polymicrobial infections: three of them presented with synovial glucose levels above the cutoff, and all of them presented with wound problems.

There are several limitations in our study that should be acknowledged. Firstly, this is a retrospective study, which inherently comes with its own limitation. Additionally, a significant number of patients had to be excluded due to missing synovial fluid data. Secondly, the lack of a gold standard for PJI diagnosis led us to adopt the 2013 MSIS criteria as our diagnostic reference; we preferred these criteria because certain tests required by more recent criteria, such as the 2018 ICM or EBJIS criteria, were not applicable to all of our patients. Lastly, our study was conducted at a single center with a relatively small sample size. Therefore, well-designed multicenter studies focusing on synovial glucose for PJI diagnosis are warranted to assess and validate the performance of this biomarker.

In conclusion, both synovial glucose and serum-to-synovial-glucose ratio demonstrated good diagnostic potential for acute postoperative PJI following TKA, with AUC values of 0.861 and 0.889, respectively. The accuracy is lower in those infections that are secondary to a wound dehiscence. Notably, these biomarkers exhibited superior accuracy compared to the combination of serum CRP, synovial WBC count, and PMN% (AUC of 0.722). In the future, it is necessary to confirm our results in larger databases including other prosthetic joints.

## Data Availability

The data used in this study are not publicly available but can be provided upon reasonable request to the corresponding author.

## Code availability

The code used for data analysis is not publicly available but can be provided upon reasonable request to the corresponding author.
